# Integrating biparametric MRI radiomics with clinical variables improves pre-treatment prediction of prostate cancer recurrence

**DOI:** 10.3389/fonc.2026.1812805

**Published:** 2026-07-15

**Authors:** Selma Bozorgpana, Indri Desiati, Mohammed R. S. Sunoqrot, Petter Davik, Guro F. Giskeødegård, Gabriel Addio Nketiah, Mattijs Elschot, May-Britt Tessem, Tone F. Bathen

**Affiliations:** 1 Department of Circulation and Medical Imaging, Faculty of Medicine and Health Sciences, Norwegian University of Science and Technology, NTNU, Trondheim, Norway; 2 Department of Radiology and Nuclear Medicine, St. Olavs Hospital, Trondheim University Hospital, Trondheim, Norway; 3 Clinic of Surgery, St. Olavs Hospital, Trondheim University Hospital, Trondheim, Norway; 4 Department of Clinical and Molecular Medicine, Faculty of Medicine and Health Sciences, Norwegian University of Science and Technology, NTNU, Trondheim, Norway; 5 Department of Public Health and Nursing, Faculty of Medicine and Health Sciences, Norwegian University of Science and Technology, NTNU, Trondheim, Norway

**Keywords:** biochemical recurrence, biparametric MRI, machine learning, prostate neoplasms, radiomics, risk prediction

## Abstract

**Introduction:**

Radiomics can quantify intratumoral heterogeneity on MRI, providing complementary information beyond clinical predictors. This study aimed to evaluate whether integrating radiomic features from pre-operative biparametric MRI (bpMRI) with standard clinical variables improves pre-treatment prediction of biochemical recurrence after radical prostatectomy. We further assessed whether radiomic features add prognostic value to established predictors and compared model performance against the D’Amico classification.

**Methods:**

In this retrospective single-center study (2015–2023), 395 men who underwent pre-operative bpMRI (3T Siemens Magnetom Skyra) before radical prostatectomy were included. Lesions were automatically detected using the in-house developed PROVIZ framework, and radiomic features were extracted from index lesions using PyRadiomics v3.1.0. A total of 153 features, including first-order, textural, shape, and anatomical descriptors, were derived from T2-weighted (T2W), ADC, and high b-value diffusion-weighted (DWI, b=1500 s/mm²) images. Clinical variables included prostate specific antigen (PSA), Gleason Grade Group (GGG), PI-RADS v2.1, clinical T stage, and age. A stacked ensemble model (Random Forest and regularized Logistic Regression as base model; Logistic Regression as meta-model) was developed using five-fold stratified cross-validation, SMOTE balancing, Optuna hyperparameter tuning, and isotonic regression-based probability calibration. Performance was evaluated using AUC, calibration, and decision-curve analysis (DCA). Prognostic value was assessed with Kaplan–Meier and Cox regression analyses.

**Results:**

The combined model achieved an AUC of 0.85 (95% CI 0.83–0.87), outperforming radiomics-only (0.78) and clinical-only (0.72) models. Calibration was strong (slope = 1.01; Brier = 0.13). DCA showed higher net benefit than D’Amico classification. High-risk patients (probability ≥ 0.26) had significantly shorter recurrence-free survival (log-rank p<0.001; HR = 5.03, 95% CI 2.7–9.9). The most influential predictors were Gleason Grade Group and PSA, together with radiomic first-order/texture features.

**Conclusion:**

Integrating bpMRI-derived radiomic features with standard clinical variables improved pre-treatment prediction of biochemical recurrence after radical prostatectomy. The combined model provided better discrimination between high-risk and low-risk recurrence groups and showed higher clinical net benefit than D’Amico. The results support the potential role of radiomics for refining individualized recurrence risk assessment in prostate cancer. External validation in independent cohorts is required before clinical implementation.

## Introduction

Prostate cancer is the most frequently diagnosed cancer in men worldwide and the fifth leading cause of cancer-related death among men globally (1, 2). Despite advances in early detection and treatment, up to 30% of patients experience biochemical recurrence (BCR) following radical prostatectomy, typically defined as a postoperative prostate-specific antigen (PSA) level ≥ 0.2 ng/mL with subsequent rises (3, 4). Early identification of men at high risk of recurrence at the time of diagnosis is critical to inform treatment planning, adjuvant therapy, and surveillance strategies (2, 5).

Several clinical risk stratification tools, including the D’Amico system, NCCN guidelines, and the Cancer of the Prostate Risk Assessment (CAPRA) score, are widely used in clinical practice (2, 6–8). These models combine prostate specific antigen (PSA), Gleason Grade Group (GGG), and clinical T (cT) stage to estimate recurrence risk but rely solely on clinical and biopsy-derived information. With the increasing adoption of MRI-targeted biopsies, variables such as the number or proportion of positive cores are becoming less reliable, as targeted sampling typically yields fewer but longer cores (9, 10). Consequently, traditional models may not fully capture the biological heterogeneity and spatial complexity of prostate cancer.

Magnetic resonance imaging (MRI) provides detailed anatomical and functional information that can improve disease localization and risk assessment. Biparametric MRI (bpMRI) is now commonly used as a streamlined alternative to multiparametric MRI, reducing scan time and cost without compromising detection of clinically significant prostate cancer (6, 48). However, conventional MRI interpretation remains largely qualitative.

Radiomics offers a quantitative approach to bridge this gap by extracting high-dimensional features from medical images to characterize tumor phenotype at a level not discernible to the human eye (11). Radiomic features derived from bpMRI describe tissue intensity, shape, and texture, capturing intra-tumoral heterogeneity and morphological complexity (12). These features may therefore complement clinical predictors by reflecting biological variations related to tumor aggressiveness and recurrence risk (13).

Recent radiomics studies have demonstrated the potential of MRI-based biomarkers for predicting treatment outcomes (13, 14). Yet, most prior work has focused on post-treatment imaging or lacked rigorous evaluation of model calibration and clinical utility at the time of diagnosis. A recent meta-analysis of 24 radiomics studies underscored these limitations, emphasizing the need for standardized validation and decision-curve assessment to evaluate clinical relevance (15). Thus, the role of MRI-based radiomics for pre-treatment recurrence prediction remains underexplored.

The present study aimed to evaluate whether integrating radiomic features from pre-operative bpMRI with established clinical variables enhances pre-treatment prediction of prostate cancer recurrence following radical prostatectomy. Specifically, we investigated the prognostic value of radiomic features in combination with PSA, GGG, PI-RADS v2.1, cT, and age, and benchmarked the resulting model against the D’Amico classification. By focusing on pre-treatment imaging, our study seeks to determine whether radiomics can provide additional prognostic value at the time of diagnosis, when decisions regarding surgery, adjuvant therapy, or surveillance are most critical. This study therefore addresses an unmet need for early, image-based recurrence prediction at the time of primary diagnosis.

## Materials and methods

### Study design and ethical approval

This retrospective single-center study was conducted at St. Olavs Hospital, Trondheim University Hospital, Norway, and approved by the Regional Committee for Medical and Health Research Ethics – Central Norway (REK 2017/576). Written information was provided to all patients, and participation was based on an opt-out consent process.

### Study population

A total of 395 male patients (mean age 65.3 ± 6.0 years, range 41.9–76.9 years) with prostate cancer who underwent robot-assisted radical prostatectomy (RARP) between March 2015 and April 2023 were included. The inclusion criteria were (i) histologically confirmed prostate cancer, (ii) available pre-operative bpMRI and (iii) core clinical features; age, PSA level (last measurement before surgery), Gleason Grade Group (GGG, from targeted biopsy), PI-RADS v2 score, and MRI-based staging (cT). Recurrence status (endpoint) was determined based on post-operative PSA follow-up, where biochemical recurrence (BCR) was defined as PSA ≥ 0.2 ng/mL followed by a subsequent rise or a persistently elevated PSA level after surgery. Lesion detection and segmentation were performed using PROVIZ, an in-house developed, fully automated deep-radiomics software for lesion-level detection of clinically significant prostate cancer (Grade Group ≥ 2) on biparametric MRI (16).

### MRI acquisition and radiomics feature extraction

All participants underwent bpMRI scanning between March 2015 and April 2023 using a Magnetom Skyra 3T MRI scanner (Siemens Healthineers, Erlangen, Germany). Image acquisition followed PI-RADS v2.1 guidelines. Detailed imaging parameters and PI-RADS scoring procedures are provided in **Supplementary Table 1**.

PROVIZ’s pipeline consists of nnU-Net-based prostate segmentation, voxel-wise feature map generation, XGBoost classification, and post-processing to detection maps (16). In this study, first-order intensity features were computed from T2-weighted (T2W), apparent diffusion coefficient (ADC), and high b-value diffusion-weighted (HBV, b = 1500 s/mm²) images. Second-order texture features (GLDM, GLRLM, GLSZM, NGTDM) and anatomical location descriptors were derived from the T2W series. For each index lesion mask (highest mean probability), the mean of each pre-computed feature map was extracted, yielding 137 radiomic features. An additional 16 3D shape features were calculated using PyRadiomics v3.1.0 (17), resulting in 153 features per lesion (see **Supplementary Table 2**).

### Machine learning model development

A machine-learning pipeline was developed to predict prostate cancer recurrence by integrating radiomic and clinical features. Data preprocessing included median imputation for missing values and standardization using StandardScaler (18). Missing values were only present in cT (N = 55). This variable was reformatted to binary (organ-confined (cT2) vs non–organ-confined (cT3) and imputed using the mode (most frequent value) from the training set.

Three feature sets were prepared for model training: one that combined both clinical and radiomic features (Combined model), one containing only radiomic features extracted from MRI scans (Radiomics-only model), and one including only clinical variables (Clinical-only model). Of the 395 included patients, 355 had at least one lesion automatically detected by PROVIZ and were therefore eligible for radiomics-based model development. The eligible cohort was randomly split into a training set (80%, N = 284) and a held-out test set (20%, N = 71) (see **Supplementary Figure 1** for details), reflecting the real-world recurrence distribution (~30 % recurrence rate) (19). From the training set, 20% (N = 57) was used for internal validation, resulting in a final training subset of N = 227 (**Table 1**). The internal validation set was used for hyperparameter tuning and overfitting prevention, while the test set was held out for final model evaluation. To address class imbalance, the Synthetic Minority Over-sampling Technique (SMOTE) was applied only on the training data to balance recurrence classes, preventing data leakage (20). To ensure robustness and account for variability in data splits, the entire modeling process was repeated five times using different random seeds. Results were aggregated across these independent runs for performance evaluation and statistical comparison.

**Table 1:** Overview of the full cohort splitting strategy.

Dataset split	Number of patients	Percentage	Recurrence/non-recurrence	
Patients Used in Model (Lesion Detected by PROVIZ)	355	100%	30/70
Training Set	284	80%	30/70
*Training Subset*	*227*	*80%*	*30/70* ➔ *50/50 after SMOTE*
*Validation Set*	*57*	*20%*	30/70
External Test Set	71	20%	30/70

Two base classifiers were used in the ensemble model: Random Forest and regularized logistic Regression (21, 22). Hyperparameter tuning was conducted using the Optuna framework (23) with five-fold stratified cross-validation (24) to optimize parameters such as the number of estimators, maximum depth, and regularization strength (see **Supplementary Table 3**).

**Figure 1:**
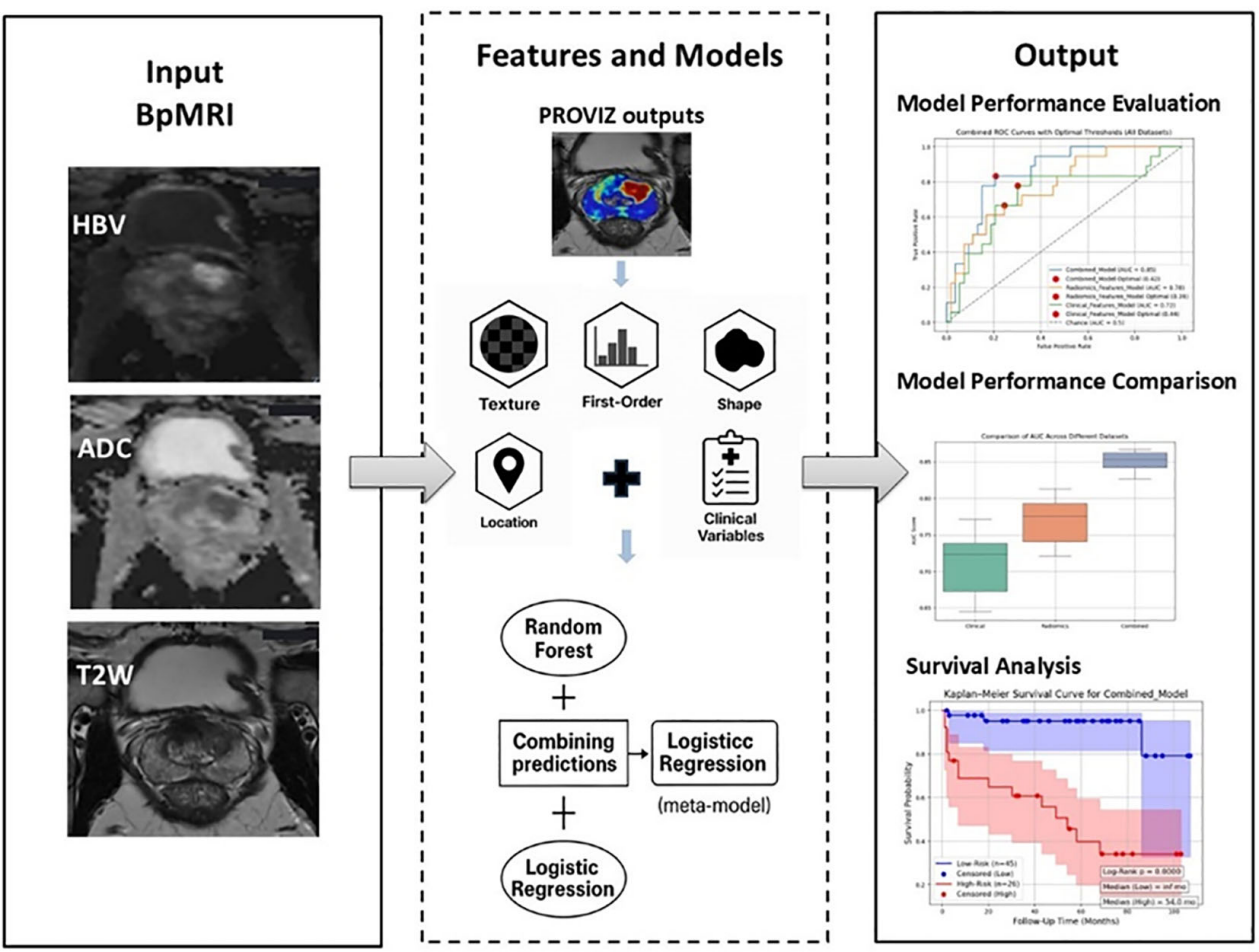
Overview of the machine-learning workflow for recurrence prediction. Schematic illustration of the pipeline integrating radiomic and clinical features for predicting prostate cancer recurrence. Biparametric MRI (bpMRI) inputs included T2-weighted (T2W), high b-value diffusion-weighted (HBV, b = 1500 s/mm²), and apparent diffusion coefficient (ADC) maps. Lesions were automatically segmented using the PROVIZ framework, and radiomic features (shape, first-order, texture, and spatial descriptors) were extracted. These features were combined with clinical variables (PSA, Gleason Grade Group, cT stage, PI-RADS, and age) to generate three datasets (clinical, radiomics, and combined). Models were trained using a stacked ensemble of random forest and logistic regression, including preprocessing steps (imputation, standardization, SMOTE), and evaluated on a held-out test set.

To combine the complementary model strengths, a stacked ensemble framework was implemented. The base models were trained on five cross-validation folds, and their predicted probabilities were used as meta-features. A regularized Logistic Regression meta-model was then trained on these aggregated predictions. Calibration performance was assessed using isotonic regression-based calibration curves, which adjust predicted probabilities to better align with observed recurrence rates. Calibration slope, intercept, and Brier score were computed to quantify agreement between predicted and observed outcomes (25, 26). The optimal decision threshold was determined from the held-out test set by minimizing the Euclidean distance to the ideal (0, 1) point on the ROC curve (27). An overview of the full method pipeline is illustrated in **Figure 1**.

### Clinical variables and risk group definitions

D’Amico risk group assignment was determined according to their published guidelines (7). We compared performance only against D’Amico, as other tools (e.g., CAPRA, NCCN) require biopsy-derived parameters (number or proportion of positive cores) unavailable in our dataset. Kaplan–Meier survival curves with log-rank testing and Cox proportional hazards models were used to assess recurrence-free survival (RFS) and estimate hazard ratios (HRs) with 95% confidence intervals (28, 29). Harrell’s concordance index (C-index) and decision curve analysis (DCA) were used to compare the discriminative performance and clinical utility of the D’Amico classification relative to the combined model (30). For comparability with the binary recurrence prediction, D’Amico categories collapsed into low/intermediate (non-recurrence) and high-risk (recurrence) groups prior to model evaluation.

### Performance evaluation and statistical analysis

Model performance was evaluated using area under the ROC curve (AUC), accuracy, sensitivity, and specificity, positive predictive value (PPV), and negative predictive value (NPV), each with 95% confidence intervals obtained from 1,000 bootstrap iterations. Confusion matrices were generated at optimal thresholds to calculate these metrics. Additional evaluation included Brier score (31), precision–recall curves, decision-curve analysis (DCA) (30), as well as calibration slope and intercept. Learning curves were used to assess model generalization across cross-validation and test sets. Feature importance was derived from Random Forest Mean Decrease in Impurity (MDI) (32).

Kaplan–Meier survival analysis was performed using predictions from the median-performing model fold (test AUC closest to median) to evaluate the prognostic value of the combined, radiomics-only, and clinical-only models. Patients were stratified into high- and low-risk groups using the validation-derived threshold (≥ 0.26 for high risk; < 0.26 for low risk). Recurrence-free survival differences were tested with log-rank tests (28). Cox proportional hazards models with 1,000 bootstrap iterations estimated hazard ratios (HR) and corresponding 95% confidence intervals (CI) (29, 33). Model discrimination was assessed using Harrell’s concordance index (C-index) (29).

Statistical comparisons between groups were performed using Mann-Whitney U tests, with normality checked via Shapiro-Wilk and D’Agostino–Pearson tests (34, 35). Multiple testing was corrected using the Benjamini-Hochberg method (α = 0.05) (36). Analyses were performed in Python (v3.7.13). We adhered to the CLEAR guidelines for transparent and reproducible radiomics research (37). Sample size calculation: Not applicable (retrospective model development on full cohort). Data overlap: No data reuse across analyses. Segmentation reliability: PROVIZ’s fully automated lesion delineations were previously validated (38), demonstrating a mean Dice coefficient of 0.84 ± 0.06 and an ICC of 0.91 for feature repeatability. Dimension reduction: Not applicable (no additional feature selection beyond ensemble tuning). Reporting standards: This diagnostic accuracy study was designed and reported in accordance with the STARD 2015 guidelines; a completed STARD checklist is provided in **Supplementary File 2**.

## Results

### Cohort characteristics

The study included 395 patients with histopathologically confirmed prostate cancer (mean age 65.3 ± 6.0 years, range 41.9-76.9 years). Of these, 355 (89.9%) patients had at least one lesion identified by PROVIZ and data from the index lesion were used in model development. Clinical characteristics stratified by PROVIZ status and recurrence status are presented in **Table 2**. All further analyses were restricted to PROVIZ-positive patients (N = 355), as radiomic features could only be extracted from detected lesions. PROVIZ-positive patients exhibited significantly higher PI-RADS scores (p < 0.001) and more radiologically distinct lesions, consistent with radiologist-observed findings. Patients who experienced recurrence had significantly higher PSA levels (p < 0.001), Gleason Grade Group (GGG; p < 0.001), and clinical T stage (p < 0.01) compared to non-recurrence patients while no significant differences were observed in age or PI-RADS between these two groups. During follow-up, 92 patients (30%) experienced recurrence over a median follow-up of 58 months (reverse Kaplan–Meier estimate).

**Table 2:** Clinical characteristics of the study cohort presented as median values across groups stratified by Proviz detection status and recurrence outcome.

Group	Number of patients (N)	PI-RADS score	cT	GGG	PSA	Age
All Patients	395	4	2	2	9.1	66
Recurrence	92	4	2*	3*	12.3*	64
Non-Recurrence	263	4	2*	2*	8.7*	65
Proviz-Positive	355	4*	2	3	9.1	65
Proviz-Negative	40	1*	2	3	7.00	63

Statistically significant differences (adjusted p < 0.05) are indicated in bold and marked with an asterisk (*).

Among the 92 patients with recurrence, the median time to recurrence was 19 months (range: 0–90 months), with 28.3% (n = 26) experiencing very early recurrence (≤3 months), consistent with persistent disease.

Bold values indicate statistically significant differences between groups (adjusted p < 0.05).

### Predictive model performance

**Figure 2:**
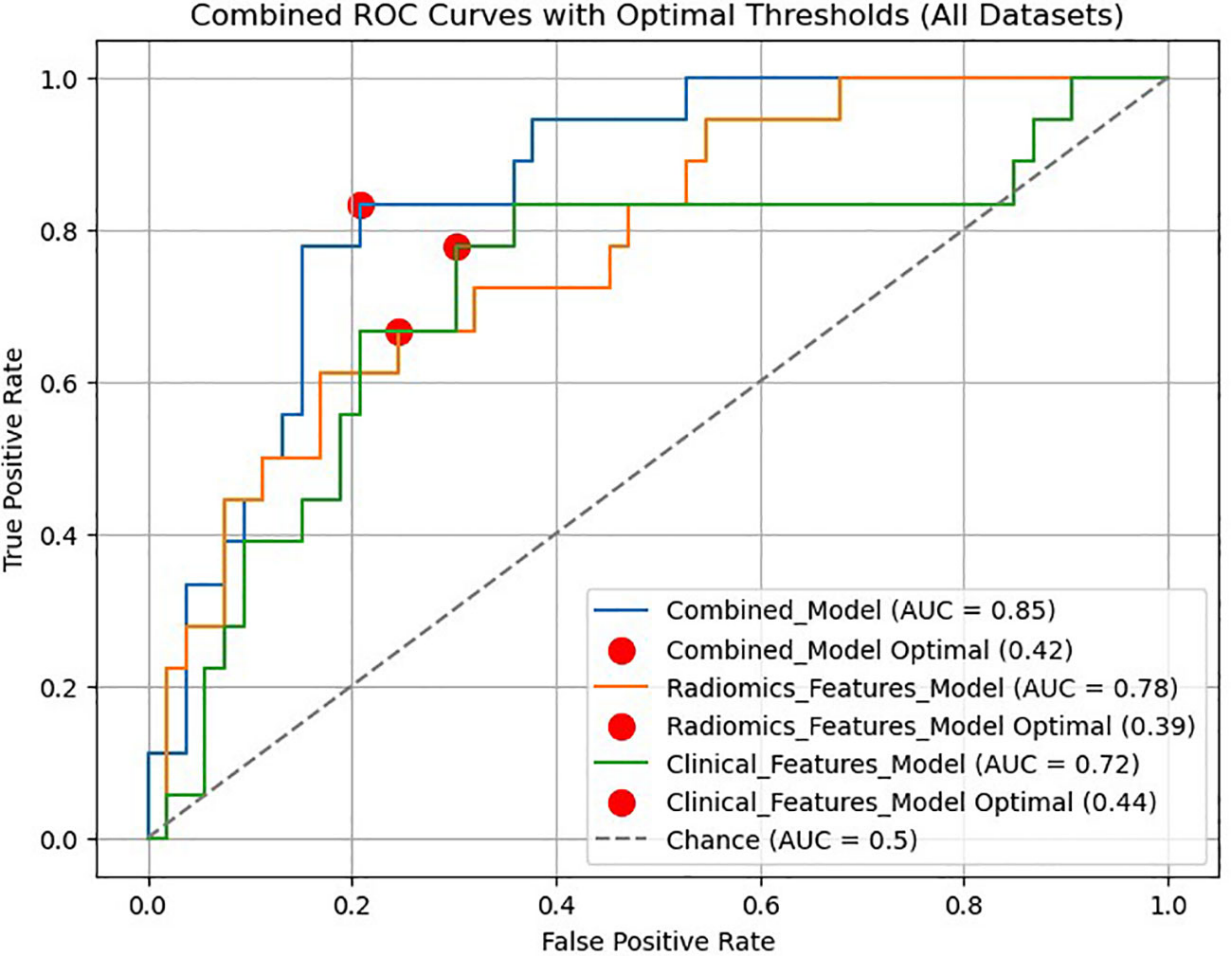
Receiver operating characteristic (ROC) curves for model comparison. ROC curves of the combined (radiomics + clinical), radiomics-only, and clinical-only models evaluated on the held-out test set (N = 71). The combined model achieved the highest AUC (0.85), outperforming both individual models (p < 0.01, DeLong test). Shaded areas represent 95% confidence intervals from 1,000 bootstrap iterations.

Model performance metrics evaluated on the held-out test set (N = 71) are summarized in **Table 3**. The combined model, integrating both radiomic and clinical features, achieved superior predictive performance (AUC = 0.85) compared to the radiomics-only model (AUC = 0.78) and the clinical-only model (AUC = 0.72) (**Figure 2**). Pairwise AUC comparisons confirmed statistically significant improvement for the combined model compared with the clinical-only (p = 0.008) and radiomics-only (p = 0.004) models after Benjamini-Hochberg correction. Using the predefined probability threshold of 0.26 derived from the validation set, the combined model achieved a PPV of 0.45 (95% CI 0.29-0.62) and a NPV of 0.95 (95% CI 0.86-1.00), based on a recurrence prevalence of 30% in our cohort. The threshold was determined exclusively from the validation data and applied unchanged to the held-out test set to avoid information leakage. Bootstrap resampling with 1,000 iterations confirmed the robustness of these estimates.

**Table 3:** Performance metrics of predictive models for prostate cancer recurrence prediction based on the median-performing fold of the test set.

Metric	Clinical model (median, 95% CI)	Radiomics model (median, 95% CI)	Combined model (median, 95% CI)
AUC	0.72 (0.65–0.77)	0.78 (0.72–0.81)	**0.85 (0.83–0.87)**
Sensitivity	0.78 (0.61–0.89)	0.67 (0.61–0.78)	**0.83 (0.78–0.89)**
Specificity	0.70 (0.66–0.76)	0.77 (0.66–0.83)	**0.79 (0.71–0.83**)
Accuracy	0.72 (0.66–0.73)	0.73 (0.69–0.77)	**0.80 (0.75–0.82)**

Pairwise AUC comparisons (adjusted p-values, Benjamini–Hochberg correction): Combined vs Clinical: 0.0076; Combined vs Radiomics: 0.0035; Clinical vs Radiomics: 0.1287.

Bold values indicate the best-performing metric among the compared models.

Confusion matrices for the models are provided in **Supplementary Figure 2**. Calibration analysis demonstrated good agreement between predicted and observed probabilities (**Supplementary Figure 3**). The precision-recall curve (AP) = 0.66 further supported stable performance across clinically relevant thresholds (**Supplementary Figure 4**).

### Decision-curve analysis

**Figure 3:**
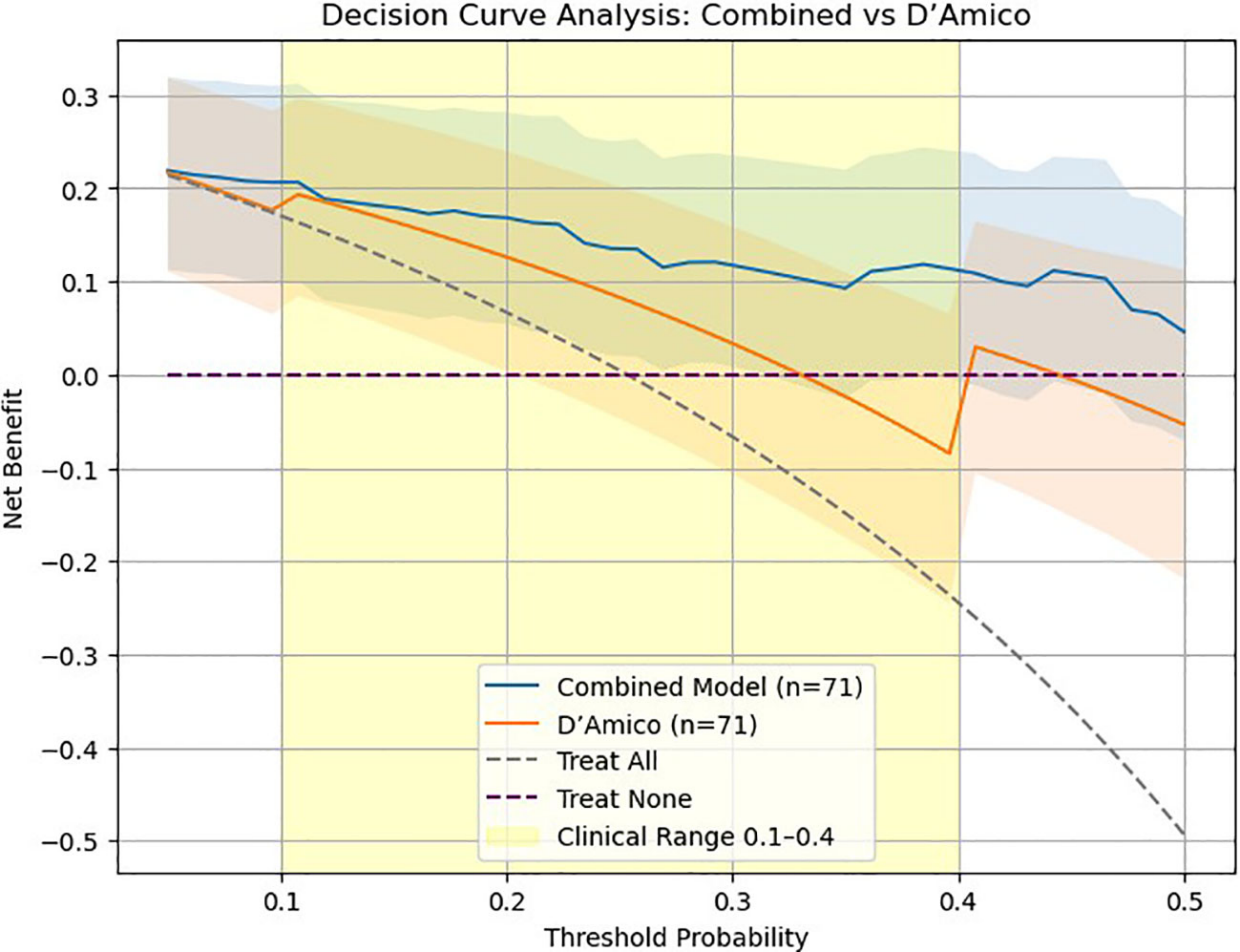
Decision-curve analysis comparing the combined model and D’Amico classification Net benefit curves across threshold probabilities (0.05–0.50). The combined radiomics–clinical model consistently outperformed the D’Amico classification and both “treat-all” and “treat-none” strategies within the clinically relevant range (0.10–0.40).

Decision-curve analysis (**Figure 3**) showed that the combined model provided a higher net benefit than the D’Amico classification across the full threshold probability range (0.05-0.50). Within the clinically relevant range (0.10-0.40), both models demonstrated positive net benefit compared to the “treat-all” and “treat-none” strategies.

### Feature importance and group-wise comparisons

In the combined model, the most important features, contributing to the prediction included GGG, PSA, and radiomic features such as the HBV_firstorder_Kurtosis and T2W_firstorder_Kurtosis. PSA and cT were also ranked highly. **Table 4** lists the top 15 features by importance in the combined model. The radiomics feature model ranked T2W_firstorder_Kurtosis, HBV_firstorder_Kurtosis, and ADC_firstorder_Skewness among its top features (**Supplementary Table 4**). In the clinical feature model, GGG, PSA, and cT remained the most influential (**Supplementary Table 5**).

**Table 4:** The 15 most important features from the combined prediction model, ranked by importance score derived from the Random Forest component (mean decrease in impurity, MDI).

Rank	Feature	Importance	Raw p-value	Corrected p-value
1	GGG	0.0426	< 0.001	0.000
2	HBV_firstorder_Kurtosis	0.0286	0.012	0.022
3	T2W_firstorder_Kurtosis	0.0241	< 0.001	< 0.001
4	T2W_firstorder_Skewness	0.0196	0.021	0.035
5	Last_PSA	0.0196	< 0.001	< 0.001
6	T3_Stadium	0.0174	0.003	0.009
7	T2_Stadium	0.0171	0.003	0.009
8	original_shape_Sphericity	0.0166	0.066	0.099
9	ADC_firstorder_Skewness	0.0146	< 0.001	< 0.001
10	ADC_firstorder_Kurtosis	0.0143	0.085	0.881
11	T2W_glcm_ClusterShade	0.0140	0.069	0.015
12	RDB	0.0140	0.389	0.449
13	Zpos	0.0128	0.152	0.190
14	original_shape_Maximum3DDiameter	0.0119	0.881	0.881
15	Prostate_Volume	0.0117	0.081	0.111

Statistical significance of differences between recurrence and non-recurrence groups was assessed using the Mann–Whitney U test with Benjamini–Hochberg correction for multiple comparisons (α = 0.05). Corrected p-values < 0.05 were considered statistically significant.

### Kaplan-Meier and Cox survival analysis

**Figure 4:**
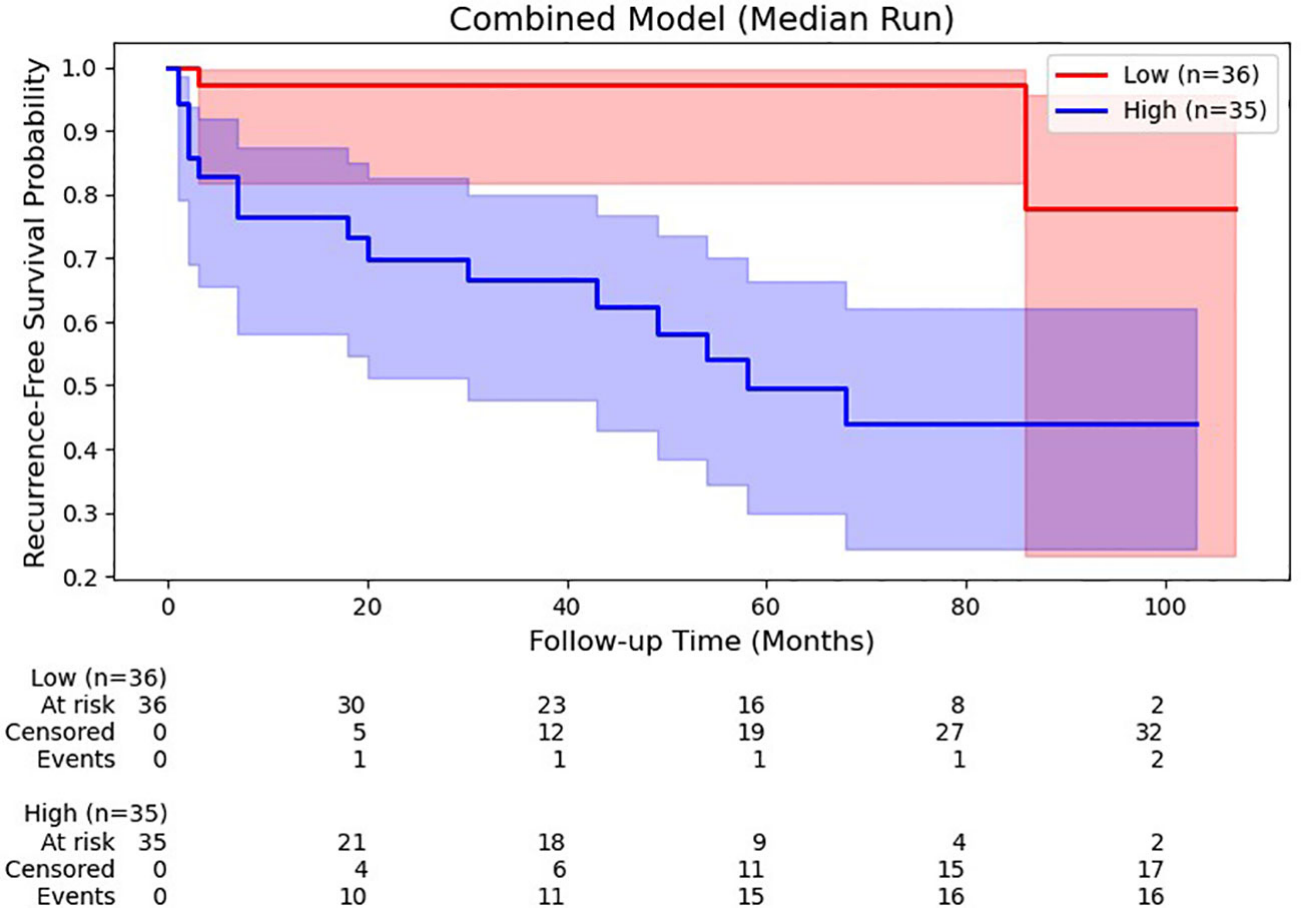
Kaplan–Meier recurrence-free survival curves for the combined model. Patients were stratified into high- and low-risk groups based on the validation-derived probability threshold (≥ 0.26 = high risk; < 0.26 = low risk). The high-risk group showed significantly reduced recurrence-free survival (log-rank p = 0.0001). Shaded areas represent 95% confidence intervals.

Kaplan-Meier survival analysis demonstrated significantly reduced recurrence-free survival among patients classified as high risk by the combined model using the validation-derived probability threshold (≥ 0.26) (log-rank p = 0.0001). The median recurrence-free survival in the high-risk group was 58.0 months, whereas the low-risk group did not reach median survival during follow-up (**Figure 4**). Cox proportional hazards regression confirmed the prognostic value of the model-derived risk stratification. Patients classified as high risk had a significantly increased risk of recurrence (HR = 5.03, 95% CI 2.67–9.86). The combined model demonstrated strong discriminative performance (C-index = 0.81) (**Table 5**).

**Table 5:** Cox proportional hazards regression analysis of recurrence-free survival for the combined radiomics–clinical model and the D’Amico classification.

Model	Comparison (reference = low risk)	HR	95% CI	P-value	C-index
Combined model	High vs Low	5.03	2.67–9.86	<0.001	0.81
D’Amico	High vs Low	3.35	1.29–8.67	0.01	0.73
	Intermediate vs Low	0.90	0.30–2.71	0.85	

For comparison with the D’Amico classification, Kaplan–Meier analysis showed significant separation between high- and low-risk groups (log-rank p = 0.0044) and between high- and intermediate-risk groups (p = 0.0091), whereas intermediate versus low-risk groups were not significantly different (p = 0.2594) (**Supplementary Figure 5**). Corresponding Cox regression results are summarized in **Table 5**. The D’Amico classification achieved a C-index of 0.73.

## Discussion

This study demonstrates that integrating radiomic features derived from bpMRI with conventional clinical features significantly improves the prediction of prostate cancer recurrence. The combined model achieved a superior AUC of 0.85, outperforming both models using clinical features (AUC = 0.72) or radiomics features (AUC = 0.78) alone. This performance gain was consistent across sensitivity, specificity, and accuracy, and was statistically significant in pairwise comparisons.

Importantly, the calibration analysis performed on the held-out test set for the combined model confirmed close agreement between predicted and observed probabilities, supporting the model’s reliability for individualized risk estimation (25, 26). The precision-recall analysis further demonstrated robust performance in identifying recurrent cases, which is particularly relevant in imbalanced clinical cohorts where positive cases are fewer (39). Together, these results highlight both the discriminative and probabilistic validity of the model’s predictions. Moreover, survival analysis confirmed the prognostic value of the predicted recurrence probabilities, with a hazard ratio of 5.03 and a concordance index of 0.81, indicating strong discriminative capability for patient risk stratification.

These findings expand upon prior work showing that radiomics can add predictive power beyond conventional clinical features (13, 14, 40, 41). In our model, key predictors included GGG, PSA and cT, well known clinical risk factors (2, 42) as well as radiomic features such as kurtosis and skewness derived from T2W, ADC, and HBV images. These quantitative imaging features may reflect aspects of tumor heterogeneity, glandular architecture, and cellularity, which are not readily captured by visual interpretation alone (12, 43, 44). However, no direct histopathological correlation analyses were performed in the present study and therefore these biological interpretations should be considered exploratory.

Notably, PI-RADS score did not demonstrate independent prognostic value in the multivariable setting. Although PI-RADS is widely used for identifying clinically significant lesions, our findings are consistent with prior work suggesting its limited utility for long-term outcome prediction when used in isolation (16, 45, 46). Radiomic features provide a more detailed and quantitative description of tumor phenotypes compared to conventional clinical variables. The radiomics model achieved a higher AUC than the clinical model, but the two models misclassified different patients. This supports the hypothesis that clinical and imaging features represent complementary, non-redundant sources of information (44). By using this complementarity, the combined model achieved more robust and generalizable prediction. Several radiomic features including HBV_firstorder_Kurtosis and T2W_firstorder_Kurtosis, were significantly associated with recurrence in univariate analysis, further reinforcing their prognostic value.

The relatively high number of predictors compared with the number of recurrence events warrants considerations. Although modern machine-learning methods can accommodate high-dimensional datasets, the possibility of residual overfitting cannot be entirely excluded. Several measures were implemented to reduce this risk, including repeated five-fold cross-validation, regularization, hyperparameter optimization, ensemble learning, calibration assessment, and evaluation on an independent held-out test set. Nevertheless, validation in larger external cohorts is required to confirm model stability and generalizability.

To contextualize performance relative to clinical practice, we compared our model against the D’Amico risk classification. Kaplan–Meier and Cox analyses confirmed that D’Amico was able to separate recurrence-free survival groups, with a C-index of 0.73 in our cohort. However, our combined model achieved higher discrimination (C-index 0.81), indicating that incorporating imaging-derived features provides complementary prognostic information beyond standard clinical parameters. Several of the top-ranked features in our model, such as PSA, Gleason Grade Group, and cT, overlapped with conventional risk factors, supporting biological consistency across methods. At the same time, radiomic features contributed additional, image-based information that complemented clinical parameters and helped refine recurrence risk estimation.

A key strength of this study is the combination of a relatively large sample size (N = 355) and an extended follow-up period (median 45 months), exceeding the duration reported in earlier radiomics studies (40, 41, 47). This strengthens the reliability of recurrence classification and outcome assessment. The use of a held-out test set that was never involved in training or tuning supports the model’s validity and guards against overfitting. Furthermore, SMOTE was applied exclusively to the training data to correct for class imbalance, while maintaining the original distribution in the internal validation and the test set ensuring fair and realistic evaluation. The stacked ensemble design, together with calibration and Cox-based survival analyses, provided converging evidence for the prognostic reliability of the predicted probabilities and the model’s clinical applicability.

In addition, decision-curve analysis (DCA) demonstrated the potential clinical usefulness of the combined model, showing a consistently higher net benefit across the clinically relevant threshold range (0.10–0.40) compared with the D’Amico classification (30). This finding underscores that our combined model can improve decision-making in postoperative management by more effectively identifying patients who may benefit from closer surveillance or adjuvant therapy, while minimizing overtreatment among low-risk cases.

Despite these strengths, several limitations should be acknowledged. The absence of external validation represents the principal limitation of this study. Although model performance remained stable across repeated training runs and an independent held-out test set, the model has only undergone internal validation. Consequently, performance in external populations remains unknown and prospective multi-center validation studies are required before clinical implementation. Only patients with PROVIZ-detected lesions were included, which could introduce selection bias and limit applicability to PROVIZ-negative patients. Because radiomic features require a detectable lesion for extraction, the present model should currently be considered applicable only to patients with MRI-visible lesions. However, this ensured consistent image quality and segmentation across all included cases, thereby supporting internal validity. Patients classified as low risk had shorter follow-up durations compared with the high-risk group, leading to earlier censoring in the Kaplan–Meier analysis. Nevertheless, consistent results across cross-validation folds and in the independent test set indicate that the model remained robust despite these constraints. While we compared our model against the D’Amico classification, future work could extend these comparisons to additional clinical tools such as CAPRA and EAU risk models to more comprehensive position radiomics within existing prognostic frameworks. Finally, prospective multi-center validation studies with independent external cohorts are essential to conform generalizability, evaluate performance across scanners and institutions, and determine clinical applicability.

In future clinical workflows, our combined radiomics-based risk score can support multidisciplinary tumor board discussion by providing individualized recurrence probabilities alongside standard clinical parameters. This will help guide decisions on adjuvant therapy and follow-up intervals, facilitating more personalized patient management.

## Conclusion

In conclusion, integrating quantitative radiomic features from pre-operative bpMRI with routinely available clinical variables significantly improved pre-treatment prediction of prostate cancer recurrence. The combined model provided better discrimination between high-risk and low-risk recurrence groups and showed higher clinical net benefit than D’Amico. These results support the potential role of radiomics for refining individualized recurrence risk assessment in prostate cancer. By capturing visible and sub-visual patterns reflecting tumor heterogeneity, radiomics enhances the stratification of patient risk providing added value when combined with established clinical predictors such as PSA, Gleason Grade Group, and MRI-based staging. Prospective multi-center studies and integration with decision-support frameworks are essential next steps to validate and implement radiomics-guided risk models in clinical workflows.

## Data Availability

The raw data supporting the conclusions of this article will be made available by the authors, without undue reservation.
