# Supplementary material for: Integrating biparametric MRI radiomics with clinical variables improves pre-treatment prediction of prostate cancer recurrence
Source: Front Oncol. 2026 Jul 15;16:1812805. doi: 10.3389/fonc.2026.1812805 (PMC13414129; doi:10.3389/fonc.2026.1812805)
Supplement: Supplementary file 1 [file DataSheet1.docx]

# Electronic Supplementary Material (ESM)

**Supplementary Materials Legend**

**Supplementary Table 1.** Summary of MRI acquisition parameters for T2‑weighted and diffusion‑weighted imaging sequences (PI‑RADS v2.1 compliance).
**Supplementary Table 2.** Categorized summary of radiomic and clinical features used for model training, grouped by feature class (FO, GLDM, GLRLM, GLSZM, NGTDM, Shape, Other).
**Supplementary Table 3.** Key hyperparameters tuned during model optimization with five‑fold stratified cross‑validation (Optuna results for combined model).
**Supplementary Table 4.** Most important radiomic features for the radiomics‑only model, with importance scores and statistical significance.
**Supplementary Table 5.** Importance rankings and statistical significance of clinical features in the clinical‑only model.

**Supplementary Figure 1.** Flowchart of patient inclusion, exclusion, and train–validation–test dataset splits.

**Supplementary Figure 2.** Confusion Matric for the combined model (median run).

**Supplementary Figure 3.** Calibration performance of prediction combined models.

**Supplementary Figure 4.** Bootstrapped precision–recall (PR) curve for the combined model

**Supplementary Figure 5.** Kaplan–Meier survival curves for D’Amico risk groups

**Supplementary Table 1.** Summary of MRI acquisition parameters for T2-weighted and DWI sequences used in the study.

| Parameter | T2-weighted (T2W) | Diffusion-weighted imaging (DWI) |
| --- | --- | --- |
| Repetition Time (TR) | 3 000–9 520 ms | 3 000–7 440 ms |
| Echo Time (TE) | ≤ 90 ms | ≤ 90 ms |
| Slice Thickness | ≤ 3 mm | ≤ 4 mm |
| In-plane Resolution | 0.47 × 0.47 mm to 0.50 × 0.50 mm | 1.42 × 1.42 mm to 2 × 2 mm |
| MRI System | 3 T Magnetom Skyra (Siemens Healthineers, Erlangen, Germany) | 3 T Magnetom Skyra (Siemens Healthineers, Erlangen, Germany) |
| b-values | – | 50, 800 s/mm² acquired; synthetic high b = 1500 s/mm² generated on-scanner |
| ADC and high-b map creation | – | ADC maps and high-b images (2 000 s/mm²) computed on-scanner |

Note: All parameters comply with PI-RADS v2.1 technical recommendations for prostate MRI (PI-RADS Steering Committee, 2019).

**Supplementary Table 2.** This table presents a categorized summary of the features extracted from bi-parametric MRI (bpMRI) scans and clinical data used in this study. Radiomic features are grouped by computational derivation: First-Order (FO) features describe intensity distributions; Gray Level Dependence Matrix (GLDM) features quantify spatial dependencies of intensity values; Gray Level Run Length Matrix (GLRLM) features capture the length of runs of similar intensities; Gray Level Size Zone Matrix (GLSZM) features evaluate regions with homogeneous intensities; and Neighboring Gray Tone Difference Matrix (NGTDM) features assess local texture complexity. Shape features describe lesion morphology. Additionally, an “Other Features” category includes anatomical location (e.g., Xpos, Ypos, Zpos), lesion probability scores (PROVIZ), and clinical parameters (PSA, Gleason Grade Group [GGG], PIRADS, and age). All features were included in model training for recurrence prediction.

| FO (First Order) | GLDM | GLRLM | GLSZM | NGTDM | SHAPE |
| --- | --- | --- | --- | --- | --- |
| ADC_firstorder_InterquartileRange | T2W_gldm_DependenceNonUniformityNormalized | T2W_glrlm_LongRunEmphasis | T2W_glszm_ZoneEntropy | T2W_ngtdm_Contrast | original_shape_Elongation |
| HBV_firstorder_RobustMeanAbsoluteDeviation | T2W_gldm_SmallDependenceLowGrayLevelEmphasis | T2W_glrlm_GrayLevelVariance | T2W_glszm_GrayLevelVariance | T2W_ngtdm_Busyness | original_shape_Flatness |
| ADC_firstorder_Kurtosis | T2W_gldm_SmallDependenceEmphasis | T2W_glrlm_ShortRunLowGrayLevelEmphasis | T2W_glszm_HighGrayLevelZoneEmphasis | T2W_ngtdm_Coarseness | original_shape_LeastAxisLength |
| HBV_firstorder_90Percentile | T2W_gldm_GrayLevelVariance | T2W_glrlm_HighGrayLevelRunEmphasis | T2W_glszm_GrayLevelNonUniformityNormalized | T2W_ngtdm_Strength | original_shape_MajorAxisLength |
| T2W_firstorder_Uniformity | T2W_gldm_LargeDependenceHighGrayLevelEmphasis | T2W_glrlm_RunLengthNonUniformityNormalized | T2W_glszm_LowGrayLevelZoneEmphasis | T2W_ngtdm_Complexity | original_shape_Maximum2DDiameterColumn |
| T2W_firstorder_Entropy | T2W_gldm_LargeDependenceLowGrayLevelEmphasis | T2W_glrlm_RunEntropy | T2W_glszm_SizeZoneNonUniformityNormalized | - | original_shape_Maximum2DDiameterRow |
| ADC_firstorder_Maximum | T2W_gldm_GrayLevelNonUniformity | T2W_glrlm_LowGrayLevelRunEmphasis | T2W_glszm_LargeAreaHighGrayLevelEmphasis | - | original_shape_Maximum2DDiameterSlice |
| ADC_firstorder_90Percentile | T2W_gldm_DependenceVariance | T2W_glrlm_ShortRunHighGrayLevelEmphasis | T2W_glszm_ZonePercentage | - | original_shape_Maximum3DDiameter |
| T2W_firstorder_InterquartileRange | T2W_gldm_LargeDependenceEmphasis | T2W_glrlm_RunPercentage | T2W_glszm_ZoneVariance | - | original_shape_MeshVolume |
| ADC_firstorder_Uniformity | T2W_gldm_HighGrayLevelEmphasis | T2W_glrlm_RunVariance | T2W_glszm_SizeZoneNonUniformity | - | original_shape_MinorAxisLength |
| ADC_firstorder_RootMeanSquared | T2W_gldm_LowGrayLevelEmphasis | T2W_glrlm_LongRunHighGrayLevelEmphasis | T2W_glszm_SmallAreaEmphasis | - | original_shape_Sphericity |
| HBV_firstorder_Entropy | T2W_gldm_DependenceEntropy | T2W_glrlm_ShortRunEmphasis | T2W_glszm_LargeAreaEmphasis | - | original_shape_SurfaceArea |
| T2W_firstorder_Energy | T2W_gldm_SmallDependenceHighGrayLevelEmphasis | T2W_glrlm_GrayLevelNonUniformityNormalized | T2W_glszm_SmallAreaLowGrayLevelEmphasis | - | original_shape_SurfaceVolumeRatio |
| T2W_firstorder_90Percentile | T2W_gldm_DependenceNonUniformity | T2W_glrlm_LongRunLowGrayLevelEmphasis | T2W_glszm_LargeAreaLowGrayLevelEmphasis | - | original_shape_VoxelVolume |
| T2W_firstorder_Skewness | - | - | T2W_glszm_SmallAreaHighGrayLevelEmphasis | - | - |
| ADC_firstorder_Minimum | - | - | T2W_glszm_LowGrayLevelZoneEmphasis | - | - |
| ADC_firstorder_10Percentile | - | - | T2W_glszm_HighGrayLevelZoneEmphasis | - | - |
| T2W_firstorder_Minimum | - | - | T2W_glszm_LargeAreaHighGrayLevelEmphasis | - | - |
| HBV_firstorder_Skewness | - | - | T2W_glszm_GrayLevelNonUniformity | - | - |
| T2W_firstorder_10Percentile | - | - | - | - | - |
| ADC_firstorder_Entropy | - | - | - | - | - |
| ADC_firstorder_Mean | - | - | - | - | - |
| ADC_firstorder_Range | - | - | - | - | - |
| HBV_firstorder_RootMeanSquared | - | - | - | - | - |
| HBV_firstorder_Kurtosis | - | - | - | - | - |
| ADC_firstorder_Median | - | - | - | - | - |
| T2W_firstorder_MeanAbsoluteDeviation | - | - | - | - | - |
| HBV_firstorder_Variance | - | - | - | - | - |
| HBV_firstorder_Mean | - | - | - | - | - |
| T2W_firstorder_Maximum | - | - | - | - | - |
| ADC_firstorder_MeanAbsoluteDeviation | - | - | - | - | - |
| T2W_firstorder_Variance | - | - | - | - | - |
| ADC_firstorder_Energy | - | - | - | - | - |
| T2W_firstorder_Mean | - | - | - | - | - |
| ADC_firstorder_Skewness | - | - | - | - | - |
| T2W_firstorder_Kurtosis | - | - | - | - | - |
| T2W_firstorder_RootMeanSquared | - | - | - | - | - |
| T2W_firstorder_RobustMeanAbsoluteDeviation | - | - | - | - | - |
| ADC_firstorder_TotalEnergy | - | - | - | - | - |
| HBV_firstorder_Minimum | - | - | - | - | - |
| T2W_firstorder_Range | - | - | - | - | - |
| T2W_firstorder_TotalEnergy | - | - | - | - | - |
| HBV_firstorder_InterquartileRange | - | - | - | - | - |
| HBV_firstorder_Median | - | - | - | - | - |
| T2W_firstorder_Median | - | - | - | - | - |
| HBV_firstorder_MeanAbsoluteDeviation | - | - | - | - | - |
| T2W_firstorder_TotalEnergy | - | - | - | - | - |
| HBV_firstorder_Maximum | - | - | - | - | - |

**Supplementary Table 3.** Summary of key hyperparameters tuned during the model optimization phase using Optuna with five-fold stratified cross-validation. Random Forest and Logistic Regression were used as base models in the ensemble, with a Logistic Regression meta-model trained on out-of-fold predicted probabilities. The values shown below correspond to the Combined Model's median run.

| Model | Hyperparameter | Optimized Value |
| --- | --- | --- |
| Random Forest | n_estimators | 297 |
|  | max_depth | 22 |
|  | min_samples_split | 2 |
|  | min_samples_leaf | 1 |
|  | class_weight | 'balanced' |
| Logistic Regression | C | 10.981880441462608 |
|  | solver | 'lbfgs' |
|  | class_weight | 'balanced' |
|  | random_state | 4325 |
|  | max_iter | 1000 |
| Stacked Meta-Model | C | 0.2582781180461323 |
|  | solver | 'liblinear' |
|  | penalty | 'l1' |
|  | random_state | 4325 |
|  | max_iter | 1000 |

**Supplementary Table 4:** This table lists the most important radiomics features for the radiomics model, along with their importance scores and statistical significance.

| Rank | Feature | Importance | Raw p-value | Corrected p-value | Significant |
| --- | --- | --- | --- | --- | --- |
| 1 | T2W_firstorder_Kurtosis | 0.0279 | 6.38e-05 | 0.000879 | True |
| 2 | HBV_firstorder_Kurtosis | 0.0251 | 0.01188 | 0.03563 | True |
| 3 | T2W_firstorder_Skewness | 0.0230 | 0.02106 | 0.05265 | False |
| 4 | original_shape_Sphericity | 0.0219 | 0.06584 | 0.1235 | False |
| 5 | Zpos | 0.0184 | 0.1517 | 0.1897 | False |
| 6 | ADC_firstorder_Skewness | 0.0175 | 0.00011 | 0.000879 | True |
| 7 | T2W_glcm_ClusterShade | 0.0162 | 0.0068 | 0.03431 | True |
| 8 | original_shape_SurfaceVolumeRatio | 0.0161 | 0.0092 | 0.03452 | True |
| 9 | ADC_firstorder_Kurtosis | 0.0159 | 0.8553 | 0.8553 | False |
| 10 | Prostate_Volume | 0.0153 | 0.0812 | 0.1355 | False |
| 11 | HBV_firstorder_Skewness | 0.0139 | 0.0575 | 0.1195 | False |
| 12 | RDB | 0.0132 | 0.3892 | 0.5308 | False |
| 13 | original_shape_Maximum2DDiameterColumn | 0.0128 | 0.6508 | 0.6973 | False |
| 14 | original_shape_MajorAxisLength | 0.0122 | 0.2604 | 0.6973 | False |
| 15 | Xpos | 0.0121 | 0.5925 | 0.6973 | False |

**Supplementary Table 5:** This table presents the clinical features importance for the clinical model and statistical significance.

| Rank | Feature | Importance | Raw p-value | Corrected p-value | Significant |
| --- | --- | --- | --- | --- | --- |
| 1 | Age | 0.2868 | 0.632 | 0.6322 | False |
| 2 | Last_PSA | 0.2759 | 4.41e-06 | 1.324e-05 | True |
| 3 | GGG | 0.2277 | 1.018e-18 | 1.527e-05 | True |
| 4 | PIRADS | 0.1621 | 0.03416 | 0.0614 | False |
| 5 | cT-II | 0.0267 | 0.003434 | 0.008585 | True |
| 6 | cT-III | 0.0208 | 0.003434 | 0.008585 | True |


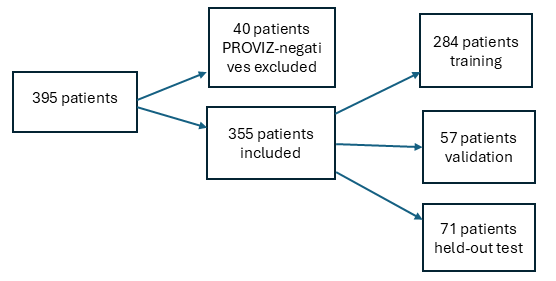


**Supplementary Figure 1.** Flowchart of patient inclusion, exclusion, and train–validation–test dataset splits.


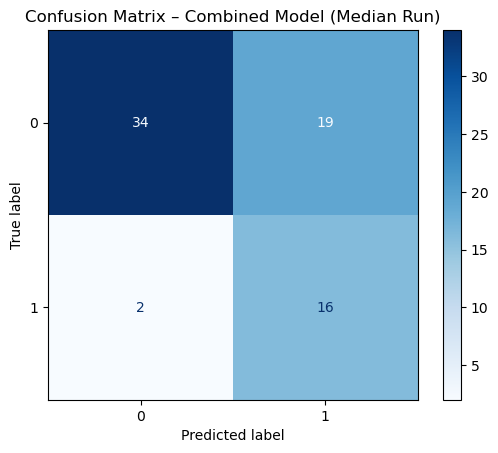


**Supplementary Figure 2.** Confusion Matric for the combined model (median run).


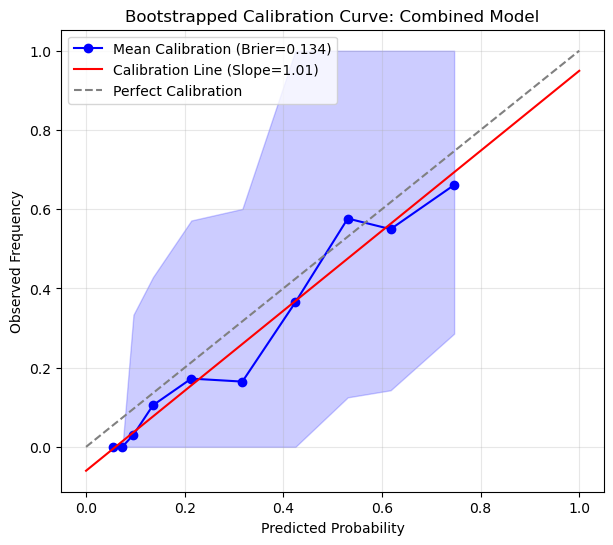


**Supplementary Figure 3.** Calibration performance of prediction combined models. Calibration curves showing the relationship between predicted and observed recurrence probabilities for the combined, radiomics-only, and clinical-only models. The combined model demonstrated the best agreement, with a calibration slope = 1.01, intercept = –0.06, and Brier score = 0.134. Shaded regions indicate 95% confidence intervals.


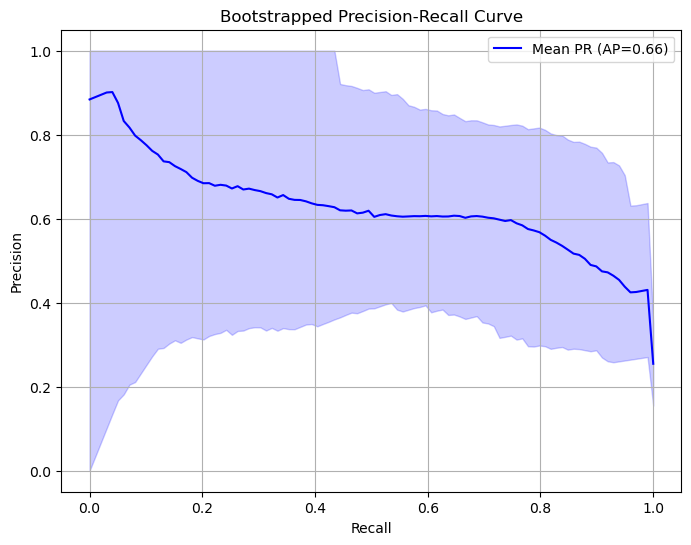


**Supplementary Figure 4.** Bootstrapped precision–recall (PR) curve for the combined model. Precision–recall curve illustrating model performance across probability thresholds. The combined model achieved an average precision (AP) of 0.66, confirming robust identification of recurrence cases in an imbalanced dataset (recurrence prevalence = 30%).


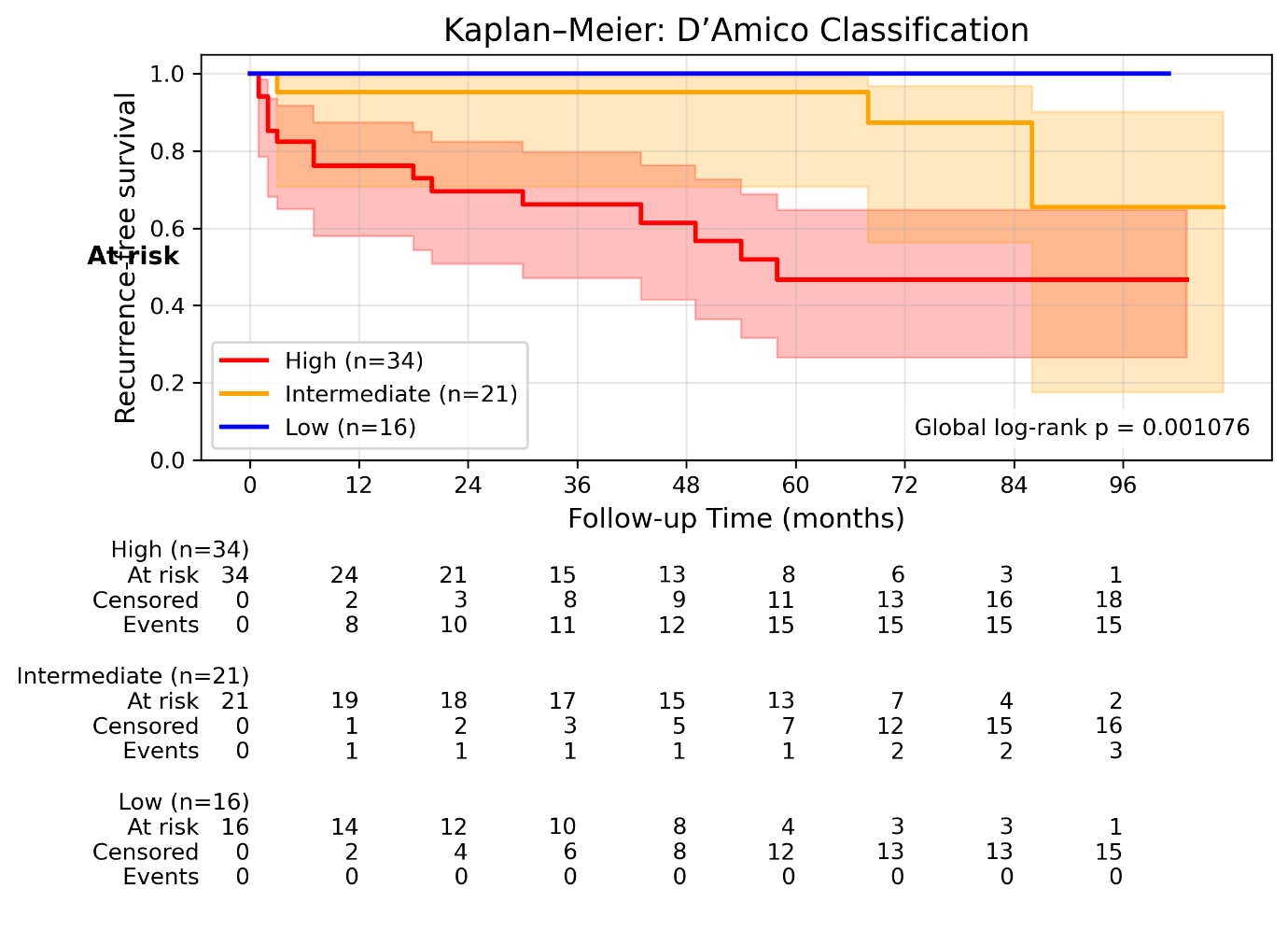


**Supplementary Figure 5.** Kaplan–Meier survival curves for D’Amico risk groups. Kaplan–Meier curves showing recurrence-free survival stratified by D’Amico risk classification (low, intermediate, high). Significant differences were observed between high- and low-risk (p = 0.0044) and between high- and intermediate-risk (p = 0.0091) groups, while intermediate vs low was not significant (p = 0.2594).
