# Supplementary material for: Integrating biparametric MRI radiomics with clinical variables improves pre-treatment prediction of prostate cancer recurrence
Source: Front Oncol. 2026 Jul 15;16:1812805. doi: 10.3389/fonc.2026.1812805 (PMC13414129; doi:10.3389/fonc.2026.1812805)
Supplement: Supplementary file 2 [file DataSheet2.docx]

| STARD Item | Description | Manuscript Location / Action Needed |
| --- | --- | --- |
| 1 | Identification as a study of diagnostic accuracy using ≥1 accuracy measure (e.g., sensitivity, specificity, AUC) | Title & Abstract: AUC, sensitivity, specificity are reported in the Abstract. ✔ |
| 2 | Structured summary of study design, methods, results, conclusions | Abstract uses Background/Methods/Results/Conclusions headings. ✔ |
| 3 | Scientific/clinical background, intended use & role of index test | Introduction describes need for pre‑treatment recurrence prediction and bpMRI radiomics role. ✔ |
| 4 | Study objectives and hypotheses | Introduction states objectives explicitly. ✔ |
| 5 | Prospective vs. retrospective design | Methods: “retrospective cohort” clearly stated. ✔ |
| 6 | Eligibility criteria | Methods: Inclusion/exclusion criteria described under “Patients.” ✔ |
| 7 | Basis for identifying potentially eligible participants | Methods: Consecutive patients undergoing radical prostatectomy in defined period. ✔ |
| 8 | Setting, location, dates of participant identification | Methods: St. Olavs Hospital, 2015–2023 specified. ✔ |
| 9 | Series type: consecutive, random, or convenience | Methods: Cohort described as consecutive. ✔ |
| 10a | Index test details, sufficient for replication | Methods: Radiomics pipeline (PROVIZ segmentation, PyRadiomics feature extraction) described. ✔ |
| 10b | Reference standard details, sufficient for replication | Methods: Biochemical recurrence (PSA ≥ 0.2 ng/mL) defined as reference standard. ✔ |
| 11 | Rationale for choosing reference standard | Methods: PSA ≥ 0.2 ng/mL is the standard definition of biochemical recurrence after prostatectomy. ✔ |
| 12a | Definition & rationale for index test cut‑off(s) | Methods: High‑risk threshold (predicted recurrence probability ≥ 0.42) described. ✔ |
| 12b | Definition & rationale for reference standard cut‑off(s) | Methods: PSA ≥ 0.2 ng/mL defined as recurrence. ✔ |
| 13a | Blinding: availability of clinical info to index test readers | Methods: Radiomic feature extraction was performed blinded to clinical outcomes. ✔ |
| 13b | Blinding: availability of index test to reference standard assessors | Not applicable, automated outcome assessment. ✔ |
| 14 | Methods for estimating/comparing accuracy measures | Methods: AUC, log‑rank, Cox regression, and C‑index described. ✔ |
| 15 | Handling of indeterminate index or reference results | Methods: No indeterminate cases, not applicable. ✔ |
| 16 | Handling of missing data | Methods: “Missing data were imputed using multiple imputation.” ✔ |
| 17 | Analyses of variability in accuracy (pre‑specified vs exploratory) | Methods: Reported bootstrapped HRs and five‑fold CV; “No pre‑specified subgroup variability analyses were performed.” ✔ |
| 18 | Intended sample size and its determination | Methods: No formal sample‑size calculation was performed due to retrospective design. ✔ |
| 19 | Participant flow diagram | Supplementary Figure 1: Flowchart of patient inclusion/exclusion and dataset splits. ✔ |
| 20 | Baseline demographic & clinical characteristics | Results/Table 1: Patient demographics and clinical variables. ✔ |
| 21a | Disease severity distribution among target‑positive | Results/Table 1: Distribution of Gleason Grade Groups. ✔ |
| 21b | Alternative diagnoses among target‑negative | Not applicable—no alternative diagnoses. ✔ |
| 22 | Time interval/clinical interventions between index test & reference | Methods: Median interval between MRI and surgery (30 days). ✔ |
| 23 | Cross tabulation of index vs. reference results | Supplementary Table 3: 2×2 contingency table for combined model added. |
| 24 | Estimates of accuracy with precision (95% CIs) | Results: AUC 0.85 (95 % CI 0.83–0.87), HRs with 95 % CIs reported. ✔ |
| 25 | Adverse events from index test or reference | Not applicable—no adverse events. ✔ |
| 26 | Limitations including bias, uncertainty, generalizability | Discussion: Limitations (single‑center, selection bias, SMOTE, follow‑up) discussed. ✔ |
| 27 | Implications for practice, intended use/clinical role | Discussion: Clinical integration paragraph added. ✔ |
| 28 | Registration number & registry name | Not applicable. ✔ |
| 29 | Where full study protocol can be accessed | Study protocol available from corresponding author. ✔ |
| 30 | Funding sources & role of funders | Declarations: Funding sources listed; added “No funder role in study design or analysis.” ✔ |
